# Dopamine D1-Receptor Organization Contributes to Functional Brain Architecture

**DOI:** 10.1523/JNEUROSCI.0621-23.2024

**Published:** 2024-02-01

**Authors:** Robin Pedersen, Jarkko Johansson, Kristin Nordin, Anna Rieckmann, Anders Wåhlin, Lars Nyberg, Lars Bäckman, Alireza Salami

**Affiliations:** ^1^Department of Integrative Medical Biology, Umeå University, Umeå S-90197, Sweden; ^2^Wallenberg Center for Molecular Medicine (WCMM), Umeå University, Umeå S-90197, Sweden; ^3^Umeå Center for Functional Brain Imaging (UFBI), Umeå University, Umeå S-90197, Sweden; ^4^Aging Research Center, Karolinska Institutet & Stockholm University, Stockholm S-17165, Sweden; ^5^Department of Radiation Sciences, Umeå University, Umeå S-90197, Sweden; ^6^Max-Planck-Institut für Sozialrecht und Sozialpolitik, Munich 80799, Germany

**Keywords:** architecture, dopamine, functional connectivity, gradients, organization

## Abstract

Recent work has recognized a gradient-like organization in cortical function, spanning from primary sensory to transmodal cortices. It has been suggested that this axis is aligned with regional differences in neurotransmitter expression. Given the abundance of dopamine D1-receptors (D1DR), and its importance for modulation and neural gain, we tested the hypothesis that D1DR organization is aligned with functional architecture, and that inter-regional relationships in D1DR co-expression modulate functional cross talk. Using the world's largest dopamine D1DR-PET and MRI database (*N* = 180%, 50% female), we demonstrate that D1DR organization follows a unimodal–transmodal hierarchy, expressing a high spatial correspondence to the principal gradient of functional connectivity. We also demonstrate that individual differences in D1DR density between unimodal and transmodal regions are associated with functional differentiation of the apices in the cortical hierarchy. Finally, we show that spatial co-expression of D1DR primarily modulates couplings within, but not between, functional networks. Together, our results show that D1DR co-expression provides a biomolecular layer to the functional organization of the brain.

## Significance Statement

Dopamine D1-receptors, the most abundantly expressed dopamine receptor type, expresses a high correspondence to the large-scale functional organization of the cortex. Differences in receptor density between unimodal and transmodal regions were related to the shape of the principal gradient of functional connectivity, contributing to greater differentiation of somatomotor and default mode networks. We also observed the covariance structure of D1-receptors to be associated with the strength of connectivity within functional networks. The discovery of a dopaminergic layer of brain organization represents a crucial first step toward an understanding of how dopamine, with close ties to behavior and neuropsychiatric conditions, potentially contribute to the emergence of functional brain organization.

## Introduction

The functional organization of the brain is assumed to be intrinsically related to cerebral microstructure. However, mapping between coordinated brain activity across distributed brain regions and their structural underpinnings have revealed a nonuniform structure-function tethering across the cortex (Zamani Esfahlani et al., 2022). This dissociation is characterized by a gradual decoupling from unimodal somatosensory regions to higher-order transmodal areas (Paquola et al., 2019; Vázquez-Rodríguez et al., 2019), aligned with models of a unimodal-to-transmodal processing hierarchy (Mesulam, 1998, 2012; Huntenburg et al., 2018). A potential mechanism mediating the dissociation between structure and function is the organization of neurotransmitter systems. In particular, neuromodulatory transmitters may change the biophysical properties of action potentials (Salinas et al., 2001; Ferguson and Cardin, 2020) to integrate neural signals across spatially segregated brain structures (Brzosko et al., 2015; Shine et al., 2021). Indeed, recent work have shown that the spatial similarity between different neuroreceptor systems covary with structural pathways and moderate couplings between structural and functional connectivity (Hansen et al., 2022). However, how the organization of individual receptor profiles may support functional architecture and modulate functional interactions is still poorly understood.

The neuromodulator dopamine (DA) plays an important role for both synaptic and neural activity (Seamans and Yang, 2004; El-Ghundi et al., 2007), and is associated with multiple physiological functions including motor control, reward mechanisms, reinforcement learning, and higher-order cognition (Abi-Dargham et al., 2002; Schultz, 2007; Robertson et al., 2015; Salami et al., 2019). Human in-vivo imaging studies have revealed that DA D1 and D2-like receptors (D1DR and D2DR) are organized by distinct subsystems, reflecting anatomical differences between dopaminergic midbrain projections (Rieckmann et al., 2011b; Papenberg et al., 2019) and functional systems (Zald et al., 2010; Papenberg et al., 2019). Moreover, recent work in nonhuman primates have revealed a gradient in D1DR density along the cortical hierarchy (Froudist-Walsh et al., 2021), characterized by greater receptor density in associative cortex compared to somatosensory cortices. This pattern mimics the principal organization of cortical function, characterized by gradual differentiation in connectivity patterns from unimodal to transmodal regions (Margulies et al., 2016; Huntenburg et al., 2018). Importantly, individual differences in D1DR and D2DR availability have been found to influence the strength of functional couplings (Rieckmann et al., 2011a; Nyberg et al., 2016; Roffman et al., 2016; Johansson et al., 2022). It is therefore likely that the spatial arrangement of DA receptors contributes to large-scale functional architecture. However, it is not known whether the D1DR system, the most abundant DA receptor, expresses similar organizational properties to the functional connectome and whether the spatial composition of D1DR modulates the topology of large-scale functional systems.

Using the world's largest combined D1DR-PET and MRI dataset to date from the DyNAMiC study (Nordin et al., 2022), we set out to test the hypothesis that regional differences in D1DR density is related to the shape of the functional connectome and modulate the strength of functional couplings. To examine the correspondence between functional and molecular organizations, we first employ a nonlinear embedding approach (Belkin and Niyogi, 2003) to decompose group representative covariance maps into a more parsimonious set of organizing principles. In this framework, functional and dopaminergic organizations are characterized as a set of low dimensional manifolds, describing transitions in covariance patterns along the cortical surface (Margulies et al., 2016; Haak et al., 2018; Mars et al., 2018; Vos de Wael et al., 2020). Next, we extend our analysis to individual participants to assay whether the regional differences in D1DR density comprising the molecular manifold accounts for inter-individual variation in functional organization, as indicated by differences in the relative position of regions in the functional manifold. Given the role of DA for functional distinctiveness (Li and Sikström, 2002), we hypothesize that individuals with greater hierarchal differentiation in D1DR density express greater bimodality of their functional gradients, with a greater range between gradient anchors. To not restrict our investigation to the low dimensional representations, we further investigate molecular-functional correspondence of regional interactions, capitalizing on discrete network boundaries. To this end, we use covariance matrices of each modality to investigate inter-regional associations between spatial D1DR covariance and functional couplings within and between canonical resting state networks.

## Materials and Methods

The current study used baseline data from DyNAMiC (Nordin et al., 2022), a prospective study of healthy individuals across the adult lifespan. The study was approved by the Swedish Ethical Review Authority, and all participants gave written informed consent prior to testing. We have reported about the study's design, imaging protocols, and procedures elsewhere (Nordin et al., 2022). Here, only methodological and material aspects of relevance for the current study is presented.

### Participants

The DyNAMiC study participants (*N* = 180) were recruited by random selection from the population registry of Umeå, Sweden, stratified by six age cohorts between 20 and 80 years (*n* = 30 per decade, 50% females). Participants were screened for a set of exclusion criteria, including contraindications to magnetic imaging, neurological disorders, brain pathology, cognitive impairment and medical conditions or treatment potentially affecting brain function and cognition. All participants completed a full set of functional and structural MRI scans, out of which 177 (*n *= 177) participants completed [^11^C]SCH23390 PET scans. Four subjects were excluded due to the following: one showed indications of subcutaneous injection during PET imaging, two were excluded due to technical problems during PET, and one participant declined to undergo PET. The final sample for the current study included 176 participants (82 females) aged 20–78 years (mean = 49, SD = 17.38).

### Imaging procedures

Magnetic resonance (MR) imaging was conducted using a 3-Tesla scanner (Discovery MR-750, General Electric), equipped with a 32-channel phased-array head coil. PET scanning was conducted using a hybrid PET/CT system (Discovery PET/CT 690, General Electric).

#### PET imaging and processing

Production of [^11^C]SCH23390 was performed by the radiochemistry laboratory of Norrlands Universitestsjukhus, Umeå University, according to procedures described previously (Nordin et al., 2022). Injections of [^11^C]SCH23390 had high molar activity and low mass [range = (205, 391) MBq, mean ± SD = 337 ± 27 MBq]. Participants were positioned in a supine position and were individually fitted with thermoplastic masks to prevent excessive head movement. Preceding the injection, a 5 min low-dose helical CT scan (20 mA, 120 kV, 0.8 s per revolution) was obtained for PET-attenuation correction. Continuous PET-measurement in list mode was initiated at the time of injection and continued for 60 min. Offline re-binning of list-mode data was conducted to achieve a sequence of time-framed data with increasing frame length: 6 × 10; 6 × 20; 6 × 40; 9 × 60; 22 × 120 s (*n* = 49 frames). Time-framed, attenuation-, scatter-, and decay-corrected PET images (47 slices, 25 cm FOV, 256 × 256-pixel transaxial images, voxel size 0.977 × 0.977 × 3.27 mm^3^) were reconstructed by using the manufacturer-supplied iterative VUE Point HD-SharpIR algorithm (6 iterations, 24 subsets, resolution recovery).

Estimation of target binding potential (BP) to nondisplaceable (BP_ND_) binding was computed with cerebellum as reference region (Johansson et al., 2022; Nordin et al., 2022). Pre-processing included frame-to-frame head motion correction and registration to T1-weighted MRI images using Statistical Parametric Mapping software (SPM12, Wellcome Institute, London, UK). Motion corrected PET data were then re-sliced to match the spatial dimensions and resolution of the MR-data (1.5 mm^3^ isotropic voxel size, 256 × 256 × 256). Partial volume effect (PVE) correction was carried out using a symmetric geometric transfer matrix (SGTM; regional correction) method implemented in FreeSurfer (Greve et al., 2016), with an estimated point-spread function of 2.5 mm full-width-at-half-maximum (FWHM). Voxel-wise BP_ND_ estimates were then computed using a simplified reference tissue model (SRTM; Lammertsma and Hume, 1996). All BP_ND_ map were subsequently normalized to a standard template using diffeomorphic anatomic registration through exponentiated lie algebra (DARTEL (Ashburner, 2007) based on subjects’ T1-weighted MRI images, spatially smoothed using a 2-mm FWHM Gaussian kernel, and affine-transformed to stereotactic MNI152 space using SPM12. Subject-specific BP_ND_ estimates were finally sampled by 400 cortical parcels (Schaefer et al., 2018). Univariate outliers in regional D1DR estimates were identified using the box plot method (1.5*IQR) stratified by age in decades and sex. Outliers were generally related to poor PET-model fit along the midline (BP_ND_ < 0.15%, 0.72% of parcels) and replaced by age-matched means for the right and left hemisphere separately.

#### MR imaging and preprocessing

High-resolution anatomical T1-weighted images were acquired by a 3D fast spoiled gradient-echo sequence. Imaging parameters were as follows: 176 sagittal slices, thickness = 1 mm, repetition time (TR) = 8.2 ms, echo-time (TE) = 3.2 ms, flip angle = 12°, and FOV = 250 × 250 mm. Anatomical T1-weighted images were used for tissue segmentation and thickness estimation using Freesurfer 6.0 (https://surfer.nmr.mgh.harvard.edu; Fischl et al., 2002), with manual corrections performed using the Voxel Edit mode in Freeview if necessary. Whole-brain functional images were acquired during resting-state while subjects were instructed to keep their eyes open, let their minds wander, and remain as still as possible. Functional images were sampled using a T2*-weighted single-shot echo-planar imaging (EPI) sequence, with a total of 350 volumes collected over 12 min. The functional sequence was sampled with 37 transaxial slices; slice thickness = 3.4 mm, 0.5 mm spacing; TR = 2,000 ms, TE = 30 ms, flip angle = 80°, and FOV = 250 × 250 mm. The functional images were further processed to reduce artefactual influence of non-neuronal sources. All images were first corrected for slice-timing differences, motion, and signal distortions. The time-series were subsequently demeaned and detrended, followed by simultaneous nuisance regression and temporal high-pass filtering (0.008 Hz) as to not re-introduce nuisance signals (Hallquist et al., 2013). Nuisance regression of physiological variables included average white matter, cerebrospinal fluid, and whole-brain time series, six motion parameters, in addition to derivatives, squares, and squared derivatives of each variable (Satterthwaite et al., 2012; Ciric et al., 2017). To further control for motion, a set of binary spike regressors were included for volumes exceeding a relative root-mean-squared displacement of 0.5 mm or framewise displacement (FD) of 0.2 (Power et al., 2012). Nuisance-regressed images were normalized to a sample-specific group template (DARTEL; Ashburner, 2007) and spatially smoothed using a 6-mm FWHM Gaussian kernel and affine-transformed to stereotactic MNI152 space. Functional connectivity graphs were subsequently created by sampling average BOLD time series from the same 400 cortical parcels used to sample D1DR BP_ND_ and labeled by network affiliation (Yeo et al., 2011). Subject-wise adjacency matrices were computed using Pearson's correlations, followed by Fisher's r-to-z transformation, and averaged across the sample for group-level analyses. The group-average graph was then transformed by an inverted hyperbolic tangent to re-normalize the range of correlation coefficients (−1 to 1) before gradient decomposition.

### Statistical analyses

#### Inter-regional correlation analysis

Between-subject Inter-Regional Correlation Analysis (IRCA; Horwitz et al., 1984) was used to investigate spatial relationships in D1DR expression. PET IRCA utilizes regional covariance in ligand uptake across subjects to characterize topological properties of molecular brain markers under the assumption that brain regions with significantly correlated binding potentials reflect biologically meaningful relationships (Horwitz et al., 1984; Veronese et al., 2019). IRCA produces robust network metrics with high test–retest reliability (Veronese et al., 2019), even with relatively modest sample sizes (Horwitz et al., 1984; Caminiti et al., 2017a). PET IRCA has previously been used to investigate metabolic connectivity (Horwitz et al., 1987, 1988, 1991; Lee et al., 2008; Di and Biswal, 2012; Morbelli et al., 2012; Sala et al., 2017; Caminiti et al., 2017b) and organization of catecholamine receptors (Cervenka et al., 2010; Zald et al., 2010; Rieckmann et al., 2011b; Tuominen et al., 2014; de Boer et al., 2019; Papenberg et al., 2019), transporters (Vanicek et al., 2017), and synthesis capacity (Cselényi et al., 2004; Verger et al., 2020). However, the method of cross-correlating ligand binding across subjects may be sensitive to inter-individual differences (Veronese et al., 2019). Therefore, to reduce ancillary covariance related to age or sex, parcel-wise binding potentials were correlated while controlling for linear and quadratic age effects, including linear effects of sex, effectively yielding a 400 × 400 population-level adjacency matrix. To evaluate the putative effect of age-related differences on inter-regional correlations, a separate matrix was computed with standardized D1DR BP values without age regression. The age-preserved and age-regressed adjacency matrices correlated highly (Spearman's Ρ = 0.786), and the age-attributable dissimilarity between the two matrices accounted for 9.2% of the total variance. This is consistent with previous reports (de Boer et al., 2019), indicating that age-related differences have a relatively small effect on inter-regional D1DR covariance.

#### Laplacian eigenmap construction

To compute gradients of D1DR and functional connectivity organization, we employed a nonlinear manifold learning technique able to identify principal gradient components. In line with previous studies (Margulies et al., 2016; Paquola et al., 2019), the functional and D1DR adjacency matrices underwent row-wise thresholding, only keeping the top 10% of positive edges. Thresholded and negative edges were set to zeros. The matrices were then converted to normalized angle matrices and decomposed using the Laplacian eigenmapping algorithm provided by the Brain Space toolbox (Vos de Wael et al., 2020) for MATLAB with default settings for the main analysis. In brief, the Laplacian eigenmapping algorithm estimates a low-dimensional embedding of the high-dimensional affinity matrices while preserving local properties in the embedded space. The locality-preserving characteristics of the Laplacian eigenmapping algorithm makes it relatively insensitive to outliers and noise compared other nonlinear manifold learning techniques (Belkin and Niyogi, 2003). The approach results in a number of eigenvectors, referred to as “gradients”. To investigate multimodal correspondences, D1DR gradients were linearly rotated using Procrustes alignment (Langs et al., 2015), a method able to resolve the order and sign of eigenvectors while preserving the structure of the manifold. Correspondence between functional and dopaminergic gradients were estimated by spatial correlations between parcel-wise gradient values (Spearman's Ρ). A set of control analyses were carried out to confirm that the size of the rotated embedding space did not yield artificially inflated correlations between modalities by using the fewest number of gradients cumulatively accounting for >50% of the total variance (*n* = 4). The reduced embedding space yielded similar associations for the three gradients considered in our main analyses, not statistically different for the first two (*p*s > 0.05), although a slightly weaker correspondence for the third gradient (*Z* = 5.16, *p* < 0.001). These results indicate that our primary results are robust to differences in the size of the embedding space, yielding a similar correspondence between the principal gradients of D1DR covariance and functional connectivity.

#### Hierarchal D1DR differences and network positions

Next, we set out to evaluate whether individual differences in D1DR distribution is related to differences in functional architecture. To quantify the degree of hierarchal distribution, as expressed by the unimodal-to-transmodal functional gradient, subject-specific D1DR maps were spatially correlated with the subjects’ functional gradients using Pearson correlation. The resulting correlation coefficient was then used to evaluate the placement of functional resting-state networks along the gradient axis. Similar to previous work (Bethlehem et al., 2020), we computed the center of mass of seven canonical resting-state networks (Yeo et al., 2011) along the functional manifold. In brief, the center of mass of each network was first computed as the median gradient value of the corresponding parcels for each network. Linear regression models where then fitted with subject-specific values for each network's center of mass, reflecting their relative position the embedding space, and D1DR-gradient correlation coefficients, including variables of age, sex, and FD as nuisance variables of no interest. Statistical significance was determined by FDR-corrected *p*-values.

#### D1DR covariance within and between functional systems

To investigate whether inter-regional D1DR covariance corresponds to the modular system-level topography of the functional connectome, we expected a difference in D1DR covariance within and between canonical resting-state networks. Moreover, we hypothesized that the topological profile of D1DRs covaries with inter-regional differences in FC and cortical thickness between functionally coupled brain regions. To test these possibilities, all cortical parcels were labeled according to their network affiliation as defined in a seven-network atlas (Yeo et al., 2011). To examine the difference in intra- and inter-modular D1DR covariance and cortical thickness, we used spatial autocorrelation-preserving permutation tests (i.e., “spin-test”; Alexander-Bloch et al., 2018). This was achieved by first creating surface-based representations of all subjects’ D1DR biding potential maps on Freesurfer's fsaverage surface, in addition to using surface-based thickness estimates from Freesurfer. Next, a spherical projection of the 400 parcel Schaefer atlas was randomly rotated 1,000 times. For each rotation, subjects’ surface based D1DR BP values were extracted for the rotated parcels and new adjacency matrices were computed as described previously. A null distribution of intra- and inter-modular edges was subsequently computed based on the surface-rotated matrices.

#### Edgewise associations between D1DR, FC, cortical thickness, and other receptor profiles

Associations between inter-regional D1DR correlations, functional connectivity, and cortical thickness were assessed by partial correlations (Spearman's Ρ) of coaxial intra- and inter-network edges, respectively, controlling for linear and quadratic effects of Euclidean distance. To this end, IRCA was used to compute a population-level cortical thickness adjacency matrix. First, parcel-wise surface-based cortical thickness estimates were adjusted for sex and linear and quadratic age effects using the method described for D1DR correlations. We further performed additional analyses to test the specificity of D1DR covariance in relation to functional connectivity by controlling for the 18 other receptor profiles available in the NeuroMaps toolbox (Markello et al., 2022), excluding D1DR. Parcellated mean receptor were weighted and spatially correlated following the method outlined in previous work (Hansen et al., 2022), yielding a receptor-similarity correlation matrix. Receptor-similarity edges were subsequently used as a covariate in addition to linear and quadratic effects of Euclidean distance. Statistical significance of partial correlations was assessed by spin-testing, randomly rotating a spherical projection of the parcellation maps 1,000 times and two-tailed statistical significance was determined at a 95% confidence level.

### Code accessibility

Neuroimaging summary data and code supporting the findings of this study are publicly available on GitHub (https://github.com/robinpedersen/D1DR_FC_Architecture). Subject-specific data is not publicly accessible due to privacy or ethical restrictions but is available upon reasonable request. Access to data by qualified investigators is subject to scientific and ethical review and must comply with the European Union General Data Protection Regulations (GDPR) and all relevant guidelines. The completion of a data transfer agreement signed by an institutional official will be required.

## Results

**Figure 1.**
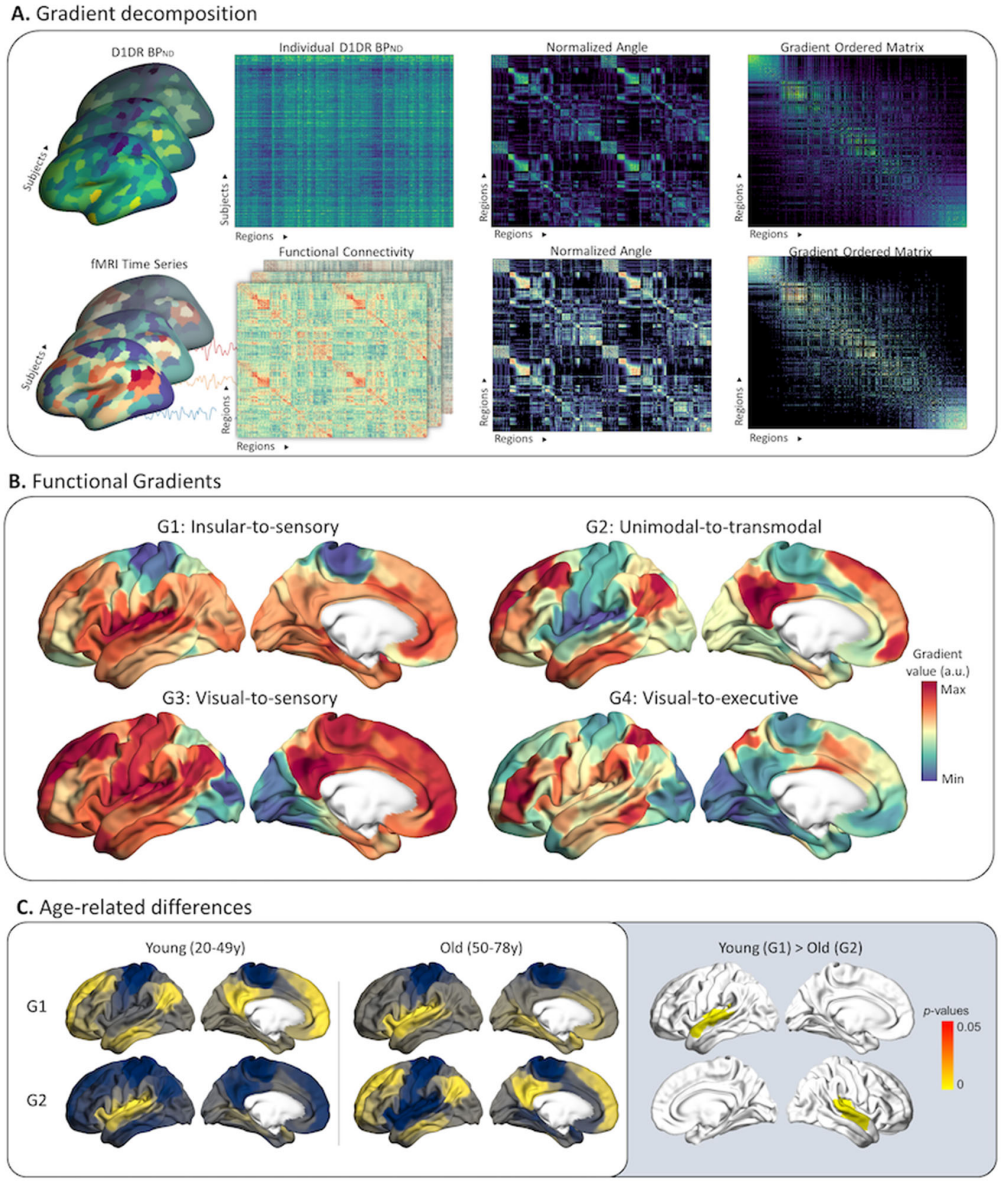
***A***, We created a group-representative cortical profile of D1DR organization and functional connectivity. Subject-specific binding potentials and rs-fMRI time series were sampled from 400 cortical parcels. Between-subject correlation analysis was used to compute inter-regional D1DR covariance, adjusted for differences in age and sex. Group-level D1DR functional connectivity matrices were converted to normalized angles and decomposed into gradients using Laplacian eigenmapping, representing spatial differences in covariance patterns. ***B***, In line with previous work, the functional gradients depicted functional differentiation along a sensory-to-insular (G1), unimodal-to-transmodal (G2), visual-to-sensory (G3), and visual-to-executive (G4) axis. ***C***, We observed group-level differences in the unimodal-to-transmodal gradient younger and older subjects. The unimodal-to-transmodal gradient explained the most variance in the younger group, followed by the sensory-to-insular gradient. The order was reversed for older subjects. Surface-based statistics revealed that the unimodal anchor of the gradient differed between the groups, located in the sensory cortex for younger adults and in the insular and superior-temporal cortex for older adults.

We constructed a cortical profile of functional connectivity and D1DR organization based on rsfMRI and [^11^C]SCH-39166 PET images from a total of 176 healthy participants (20–78 years of age) using an Laplacian eigenmapping (Belkin and Niyogi, 2003). In brief, Laplacian eigenmapping is a nonlinear manifold algorithm able to resolve low-dimensional representations of spatial connectivity patterns, commonly referred to as gradients. To this end, normalized functional connectivity matrices of 400 contiguous cortical parcels (Schaefer et al., 2018) were decomposed into Laplacian eigenvectors. In contrast to discrete functional networks, gradients provide a spectrum-like view of brain function where eigenvector values denote relative positions of parcels in the embedding space, and distances between parcels reflects similarity in covariance patterns (Fig. 1*A*). The first four functional gradients explained 60.14% of the total variance, dropping to <10% for each subsequent gradient. The first functional gradient (G1) depicted a sensory-to-insular axis of differentiation in connectivity, corresponding to the first-order gradient previously described in a separate aging sample (Bethlehem et al., 2020). The second, third, and fourth gradients depicted differentiation in connectivity from unimodal-to-transmodal (G2), visual-to-sensory (G3) and visual-to-executive (G4) regions (Fig. 1*B*), similar to the convention set by previous work (Margulies et al., 2016; Huntenburg et al., 2017; Shafiei et al., 2020). To investigate the apparent differences in gradient characteristics and order of appearance in our data and other aging samples (e.g., Bethlehem et al., 2020) to those reported in younger adults (Margulies et al., 2016; Huntenburg et al., 2017; Shafiei et al., 2020), we subsequently split the sample into a younger (20–49 years) and older (50–78 years) group. We observed that the unimodal-to-transmodal gradient explained the most variance (18.08%) in the younger group, followed by the insular-to-sensory gradient (17.65%). In reverse order, the insular-to-sensory gradient explained the most variance in the older group (20.27%), followed by the unimodal-to-transmodal gradient (17.65%). Differences were also present in the group-specific unimodal-to-transmodal gradients, with a significant cluster in the insula and superior temporal lobe, reflecting a difference in unimodal anchor between younger and older individuals (RFT-corrected *p *< 0.001; Fig. 1*C*). We did not observe any age-related differences in gradient-anchors for the remaining gradients. These findings likely explain the discrepancies in gradient characteristics in previous similar aging samples (Bethlehem et al., 2020) to those reported in younger subjects (e.g., Margulies et al., 2016).

### D1DR co-expression shares organizational principles with functional architecture along a unimodal–transmodal axis

To characterize the cortical organization of D1DR, a group-level inter-regional covariance matrix was computed as linear correlations of parcel-wise BP_ND_ estimates across subjects, adjusted for individual differences in age and sex. Using the Laplacian eigenmapping technique, we subsequently decomposed the D1DR covariance matrix into a set of group-level cortical gradients. To evaluate the specificity of D1DR in relation to functional organization, group-level intra-regional correlations in cortical thickness were decomposed and aligned following the same procedure described for D1DR and used as a covariate in a separate set of analyses. Given that the order and direction of eigenvectors may differ between modalities, Procrustes alignment (Langs et al., 2015) was used for multimodal comparisons (Vos de Wael et al., 2020). Procrustes alignment is a method able to linearly rotate the D1DR embedding space to the group-level representation of the functional data. Given sufficiently similar embedding spaces, alignment may resolve the order and direction of eigenvectors between modalities while preserving the manifold structure (Coifman and Hirn, 2014; Vos de Wael et al., 2020).

**Figure 2.**
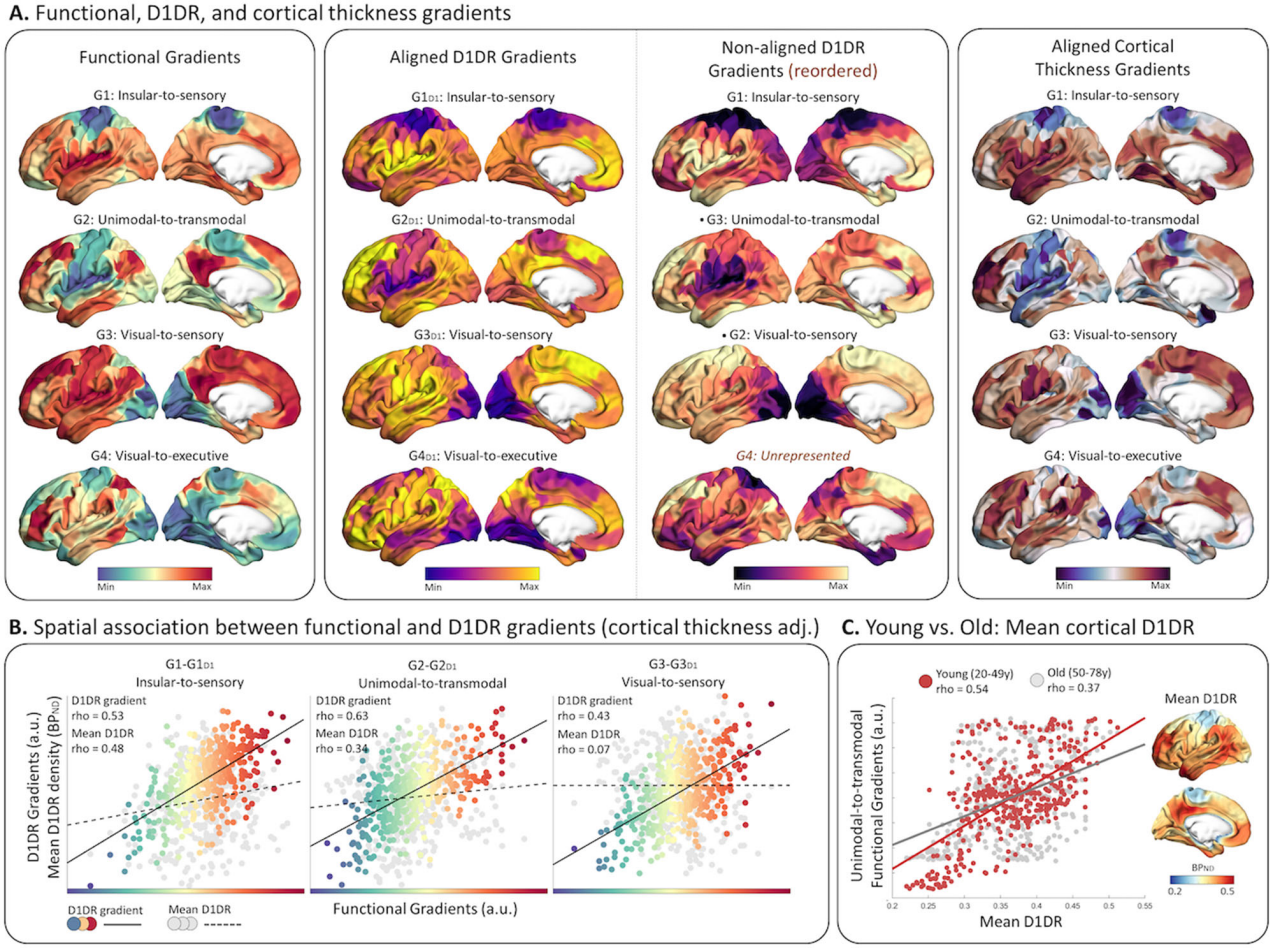
***A***, We found a spatial D1DR covariance profile similar to the organization of functional connectivity. Aligned and nonaligned D1DR gradients expressed similar topography and spatial associations to functional brain gradients G1–G3. Note that nonaligned gradients appear in a different order. Associations between functional connectivity and D1DR covariance were adjusted for cortical thickness, expressing a similar gradient profile to both functional and D1DR. ***B***, We observed a high spatial correspondence between functional connectivity and D1DR covariance gradients (bold lines). The correspondence for the unimodal–transmodal (G2) gradient was significantly between functional connectivity and D1DR compared to G1 and G3. Mean D1DR distribution largely followed the G1 and G2 axes (dashed lines). ***C***, Mean D1DR distribution expressed a greater correspondence with the unimodal–transmodal profile in younger adults compared to older adults.

The first two D1DR gradients (G1_D1_, G2_D1_) revealed a highly similar topography to the group-level sensory-to-insular functional gradient (G1) (Ρ = 0.79, *p*_spin_ < 0.001) and unimodal-to-transmodal gradient (G2) (Ρ = 0.78, *p*_spin_ < 0.001), depicting differentiation in D1DR between sensory, insular, and default-mode regions (Fig. 2). The third and fourth D1DR gradients (G3_D1_, G4_D1_) expressed a moderately strong correspondence to the visual-to-sensory (G3) (Ρ = 0.59, *p*_spin_ < 0.001) and visual-to-executive control (G4) (Ρ = 0.47, *p*_spin_ = 0.003) gradient, respectively. Importantly, when controlling for cortical thickness, the correspondence between the unimodal-to-transmodal gradients (G2-G2_D1_; Ρ = 0.63, *p*_spin_ < 0.001) was significantly greater (*Z*_diff_, *p*s < 0.03) than associations between G1-G1_D1_ (Ρ = 0.53), G3-G3_D1_ (Ρ = 0.46), and G4-G4_D1_ (Ρ = 0.43) (Fig. 2*B*). For comparison, we extended our analysis to evaluate associations without alignment. The first three nonaligned D1DR gradients were visually and statistically similar to their aligned counterparts (*p*_spin_ < 0.001), although appearing in a different order (Fig. 2*A*). The fourth nonaligned gradient was not representative of any of the four first aligned gradients and therefore omitted from further analyses. Pair-wise associations between functional and nonaligned D1DR gradients, while controlling for cortical thickness, remained significant for the unimodal–transmodal gradients (G2-G2_D1_; Ρ = 0.48, *p*_spin_ < 0.001), whereas associations between G1-G1_D1_ (Ρ = 0.42, *p*_spin_ = 0.047) and G3-G3_D1_ (Ρ = 0.28, *p*_spin_ = 0.07) were not deemed statistically significant. Next, we evaluated the spatial association between functional gradients and average D1DR distribution. Both the sensory-to-insular (G1) and unimodal-to-transmodal (G2) functional gradients expressed moderate associations with D1DR distribution (G1, Ρ = 0.48, *p*_spin_ = 0.035; G2, Ρ = 0.34, *p*_spin_ = 0.018), while no association was observed for G3 and G4 (*p*_spins_ > 0.05). Given the difference between unimodal-to-transmodal gradients in younger and older subjects, we extended our analysis to the younger and older subgroups. We found a significantly greater association (*Z*_diff_ = 2.88, *p *= 0.004) for the unimodal–transmodal gradient in younger subjects (Ρ = 0.54, *p*_spin_ < 0.001) compared to older subjects (Ρ = 0.37, *p*_spin_ < 0.001) (Fig. 2*C*). Notably, the association between D1DR distribution and the unimodal–transmodal gradient in younger subjects was on par with a corresponding gradient derived from a separate sample (Ρ = 0.54, *p*_spin_ < 0.001) (Margulies et al., 2016). Together, these results are in line with our hypothesis, suggesting a dopaminergic organization aligned with a unimodal-to-transmodal functional hierarchy, both in terms of receptor covariance and density distribution, corroborating previous reports of a unimodal–transmodal axis in D1DR density in nonhuman primates (Froudist-Walsh et al., 2021).

### Individual differences in D1DR distribution are associated with differences in functional topography

**Figure 3.**
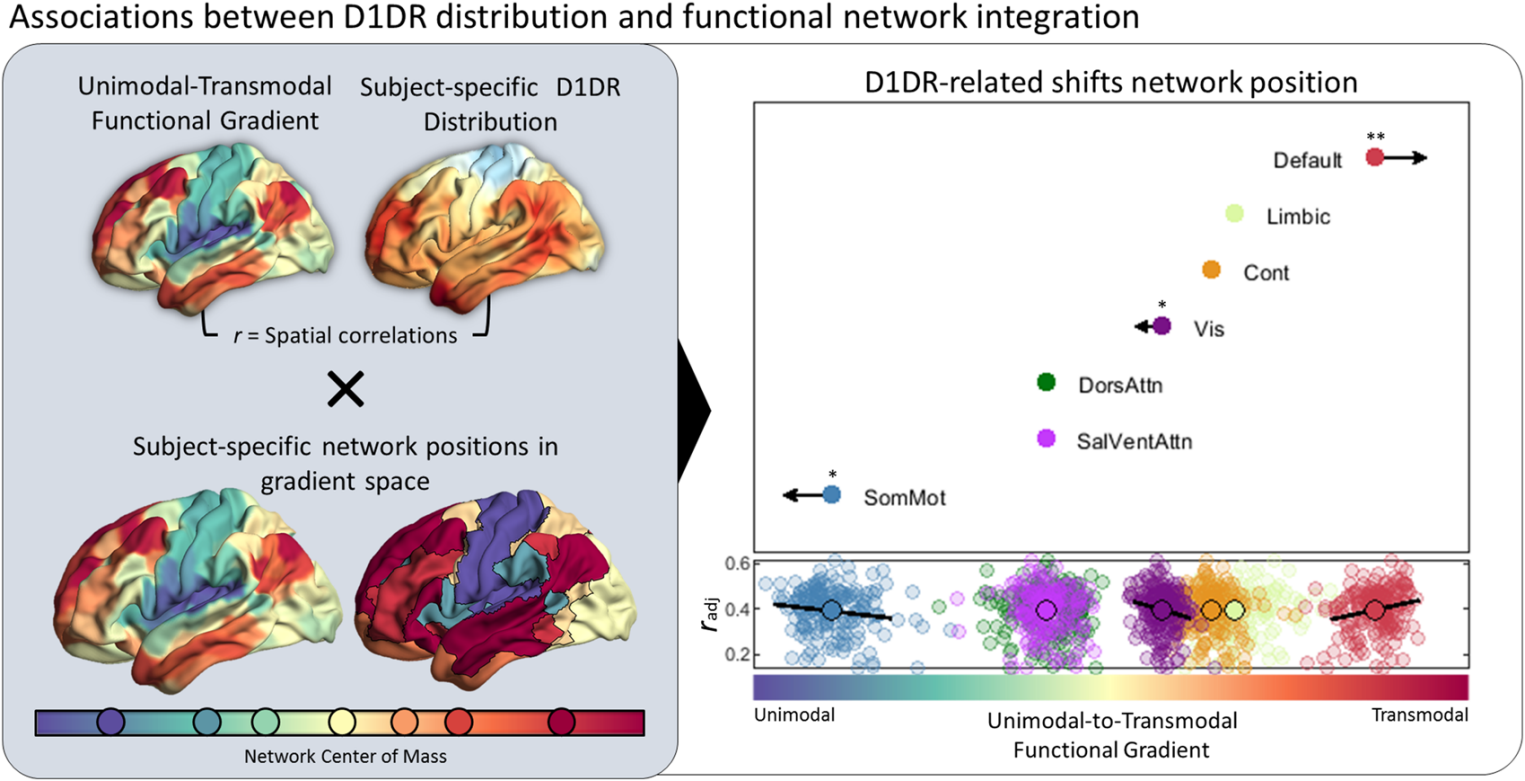
(Left) Spatial correspondence between the functional unimodal-to-transmodal gradient and subject-specific D1DR BP_ND_ maps was evaluated in relation to individual differences in network positions along gradient space. (Right) A set of multiple regression models revealed that D1DR distributions aligned to the unimodal-to-transmodal axis are associated with more unimodal positions of the somatomotor and visual network, and a more transmodal position of the default mode network. Arrows indicate directions and effect sizes for D1DR-related shifts in network position. The scatter plot reflects spatial correlations between subject-specific D1DR distributions and unimodal–transmodal gradient (*y*-axis) and subject-specific network positions along the unimodal-to-transmodal axis (*x*-axis). Axes are adjusted for effects of age, sex, and framewise displacement. SomMot, somatomotor; SalVentAttn, salience/ventral attention; DorsAttn, dorsal attention; Vis, visual; Cont, frontoparietal control; Default, default mode network. *p < 0.05; **p < 0.01.

Given the observation that cortical D1DR primarily is distributed along a unimodal-to-transmodal axis, we next sought to investigate whether individual differences in D1DR distributions are related to differences in functional architecture. We hypothesized that a more pronounced hierarchal composition of DA receptors, i.e., greater differentiation in D1DR density along the functional manifold, will manifest greater bimodality in functional organization. To this end, we computed the spatial correlation between each subject's D1DR density map and the unimodal-to-transmodal functional gradient, such that a greater correlation coefficient reflects a more pronounced unimodal-to-transmodal distribution in D1DR density. Next, the subject-specific spatial correlations between D1DR and functional gradients were used as an independent variable in a multiple regression model, testing the association with the relative position of resting-state networks in the functional embedding space (Fig. 3). We found that a more pronounced unimodal-to-transmodal distribution in D1DR was related with more a transmodal position of the default-mode network (*T* = 3.02, *p* = 0.003), and more unimodal positions of somatomotor (*T* = −2.11, *p* = 0.036) and visual (*T* = −2.45, *p* = 0.014) networks. This implies that a hierarchal composition of cortical D1DR, with greater differentiation between unimodal and transmodal cortical regions, is coupled with greater segregation between the corresponding functional networks.

### D1DR organization is related to functional connectivity strength

**Figure 4.**
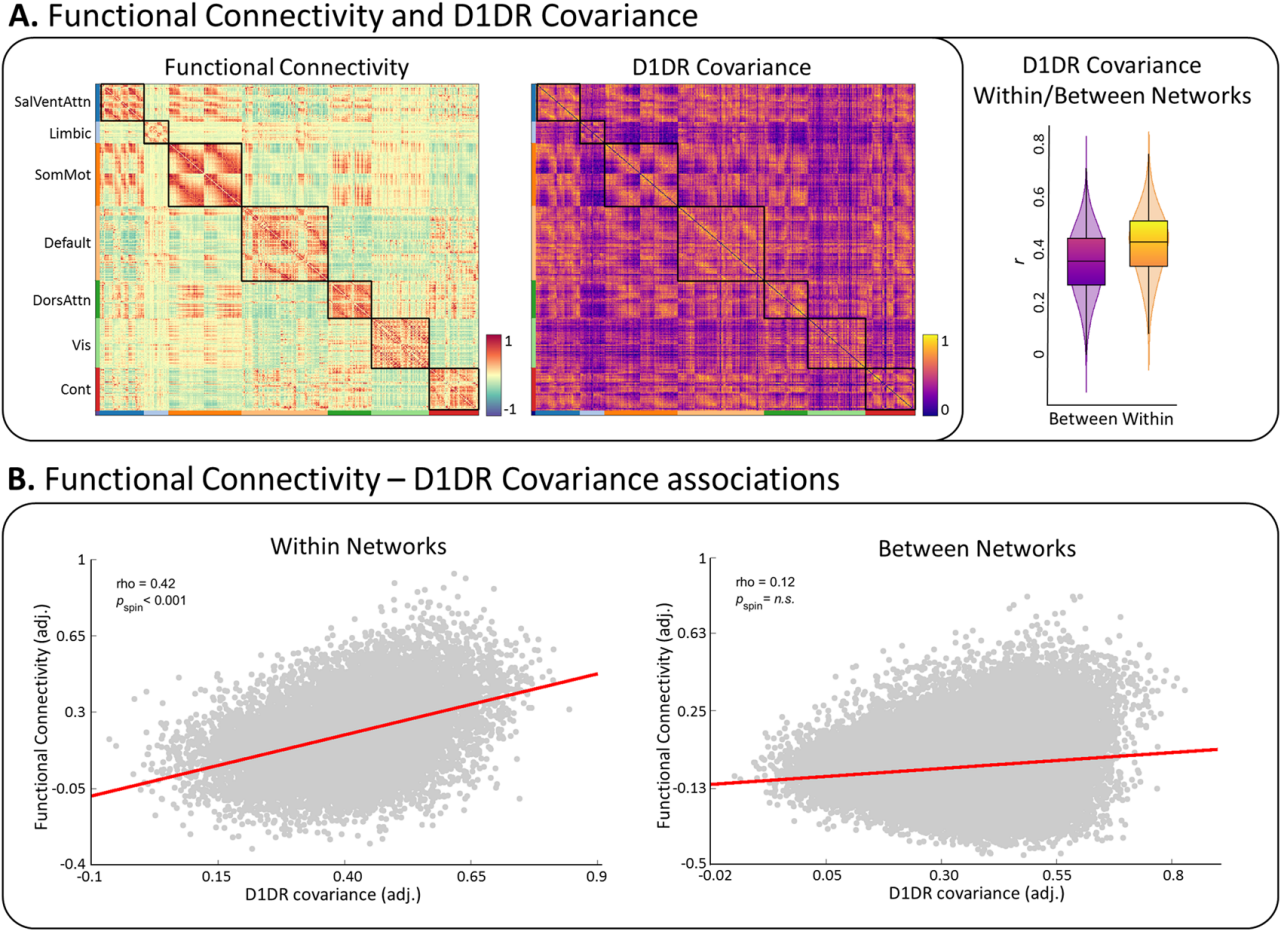
***A***, Group-level functional connectivity and D1DR covariance matrices. Borders of functional brain networks are outlined along the diagonal for visualization. We found that inter-regional D1DR covariance was significantly greater within functionally demarcated networks compared to covariance between networks. ***B***, Edgewise associations between D1DR covariance were associated with the corresponding functional connections within functional networks, but not connections between networks.

We have demonstrated that inter-regional covariance in D1DR exhibits a topographically similar organization to functional brain architecture across unimodal and transmodal regions and functional subsystems. However, a topographical overlap does not inform whether inter-regional similarity in D1DR expression modulates the strength of functional connections. We therefore evaluated the relationship between D1DR covariance and functional network organization (Yeo et al., 2011). Mean D1DR covariance was greater within networks (mean ± SD, 0.42 ± 0.13) than between networks (mean ± SD, 0.35 ± 0.13) (*p*_spin_ < 0.001), with greater in nodal density in associative networks than sensory and limbic networks (*F* = 175.72, *p* < 0.001) (Fig. 4*A*). Next, we evaluated whether the inter-regional D1DR covariance is associated with the strength of corresponding functional connections, controlling for linear and quadratic effects of Euclidean distance. Inter-regional D1DR covariance was associated with the strength of functional connectivity within networks (*r* = 0.42, *p*_spin_ = 0.01), but not between networks (*r* = 0.12, *p*_spin_ = 0.69) (Fig. 4*B*). To further examine these differences, between-network D1DR covariance was thresholded to closely match the mean covariance within networks (fraction of edges removed: 27.87%). However, thresholding did not significantly alter the within-network association between D1DR and FC (*Z*_diff_ = 0.16, *p* = 0.98).

Given that recent findings suggest that inter-regional similarity between different neuroreceptor distributions exhibit a similar association with functional connectivity (Hansen et al., 2022), we performed an additional test by controlling for the inter-regional similarity between 18 other receptor and transporter markers (Hansen et al., 2022; Markello et al., 2022). The association between D1DR covariance and intra-network FC remained significant (*r *= 0.36, *p*_spin_ = 0.013), although slightly weaker when controlling for spatial similarity of various neurotransmitters (*Z*_diff_ = 4.93, *p* < 0.001). These results indicate that inter-regional similarity of D1DR, the most abundant DA receptor in the cortex, is aligned with functional network organization and associated with stronger functional connectivity within intrinsic functional systems, independent of spatial proximity, covariance magnitude, or spatial profile of other neurotransmitter markers.

### D1DR and morphological organization uniquely contribute to functional architecture

The observed association between D1DR covariation and connectivity may reflect a common dependence on inter-regional differences in brain morphology (e.g., cortical thickness) (Smith et al., 2019; Luo et al., 2020; Garzón et al., 2021; Manninen et al., 2021). We therefore assessed the unique and shared covariance between functional connectivity and interregional covariance in relation to cortical thickness. In line with previous reports (Alexander-Bloch et al., 2018; Smith et al., 2019; Luo et al., 2020), mean cortical thickness covariance was higher within functional brain networks (mean ± SD, 0.21 ± 0.1) than between networks (mean ± SD, 0.17 ± 0.9) (*p*_spin _< 0.001). Similar to the pattern observed for D1DR, the magnitude of inter-regional covariance in cortical thickness was associated with the strength of functional connections within networks (*r* = 0.36, *p*_spin _= 0.002), but not between networks (*r *= 0.06, *p*_spin _= 0.69). Interestingly, cortical thickness and D1DR covariance expressed a moderate association both within (*r* = 0.39, *p*_spin _= 0.001) and between (*r* = 0.35, *p*_spin _< 0.001) networks, although different in effect size (*Z*_diff_ = 3.74, *p* < 0.001). An omnibus *F* test confirmed a marginal effect of both D1DR (*R*^2^_adj_ = 0.14, *p* < 0.001) and cortical thickness (*R*^2^_adj_ = 0.13, *p* < 0.001) on intra-network connectivity, respectively, with only partial mediation of cortical thickness on the association between D1DR and FC (*R*^2^_adj_ = 0.08, Sobel test: *p* < 0.001). These results suggest a spatially homogeneous interdependence between D1DR availability and cortical thickness, with unique contributions to functional intra-network connections.

## Discussion

In this work, we investigated the organization of cortical D1DR density and its relationship to functional brain architecture. We found the topographical distribution of receptors and spatial covariance pattern to follow similar organizational principles to functional brain architecture (Margulies et al., 2016). Specifically, we found a unimodal-to-transmodal hierarchy in D1DR density across the cortex, congruent with recent autoradiography findings in nonhuman primates (Froudist-Walsh et al., 2021). This is the first time a unimodal-to-transmodal distribution of D1DRs has been demonstrated in humans, expanding upon previous descriptions of the D1DR system (Seeman et al., 1987; Seamans and Yang, 2004; Karrer et al., 2017). Importantly, the unimodal-to-transmodal axis have previously been proposed as the principal processing hierarchy (Mesulam, 1998, 2012), and further analysis revealed that the covariance structure of D1 receptors expressed greater correspondence to the unimodal-to-transmodal gradient of functional connectivity compared to lower-order gradients (Margulies et al., 2016; Shafiei et al., 2020). The discovery of a hierarchal D1DR gradient along cortical mantle may thus serve as a major anatomical basis by which DA modulates functional cross talk, and in turn, relate to higher cognitive function. Importantly, we discovered that individuals with more pronounced unimodal–transmodal receptor hierarchies exhibited greater functional differentiation between somatomotor and the default mode network. This is of particular importance, given that the degree of differentiation between gradient apices has been shown to decline with age and account for differences in cognitive function (Bethlehem et al., 2020). Taken together, our findings provide a strong support for a dopaminergic layer of brain organization, contributing to the shape of macroscale functional architecture.

Given the importance of dopaminergic modulation for inter-regional signal propagation and neural gain (Seamans and Yang, 2004; El-Ghundi et al., 2007), an important question is whether the chemoarchitectural organization of D1DR receptors is associated with the strength of functional couplings. To this end, we utilized a more traditional arealization approach to investigate the correspondence between inter-regional covariance in cortical D1DR density and functional connectivity. We found that the degree of D1DR covariance was significantly greater within functional brain systems compared to between systems. Critically, region-to-region covariation in receptor density was associated with the strength of functional connections within, but not between, functionally specialized subsystems. This suggests that the chemoarchitectural profile of inter-regional associations in D1DR expression is systematically aligned with functional network structure and regulate inter-regional communication. However, recent findings suggest that spatial co-expression of different receptor types is similarly coupled with the strength of functional connections (Hansen et al., 2022). While our findings are congruent with this observation, we found a unique contribution of D1DR for intra-network connectivity, independent of other receptor profiles. Notably, our measure of D1DR covariance reflects inter-regional co-expression rather than similarity between different receptor types. This is a notable difference given that different neuroreceptor profiles are likely related with distinct properties of brain function. Moreover, both DA receptor density and functional connectivity has been linked to regional gray-matter differences (Smith et al., 2019; Luo et al., 2020; Garzón et al., 2021; Manninen et al., 2021). It is therefore likely that inter-regional covariation in D1DR availability is dependent on variation in cortical morphology (e.g., differences in shape, folding, and depth). Indeed, we found that region-to-region covariation in D1DR expression correlated strongly with covariation in cortical thickness, both within and between functional subsystems. Critically, both D1DR and cortical thickness uniquely contributed to the strength of functional connections within intrinsic resting-state networks. This suggests a functional dependency on distinct properties in both cortical gray-matter organization and dopaminergic receptor structure. The fact that brain regions exhibiting similar cortical morphology covary in D1DR availability points toward a structurally and chemoarchitecturally constrained axis of variability. Importantly, this axis largely overlaps with specialized functional subsystems and contributes to inter-regional signaling. This observation is in line with previous findings of structural covariance in cortical gray matter (Smith et al., 2019; Luo et al., 2020), suggesting that shared properties in cytoarchitectonic, chemoarchitectural, and structural covariance contribute in shaping functional brain organization (Alexander-Bloch et al., 2013; Beul et al., 2015; Smith et al., 2019; Luo et al., 2020; Valk et al., 2020; Genon et al., 2021; Shine et al., 2021).

In summary, our results constitute an important step toward an understanding of dopamine D1 system organization and its role for functional brain architecture. We expand upon previous work by highlighting the importance of inter-regional relationships in D1DR expression, beyond the topographical profile of receptor availability and anatomical gray-matter properties, in shaping the functional architecture of the brain. The discovery of a dopaminergic layer of functional brain organization represents a crucial first step toward an understanding of how DA, with close ties to behavior and neuropsychiatric conditions, potentially contribute to the emergence of functional brain organization. Understanding the dopaminergic layer of functional organization will provide a potential modifiable target to ameliorating and delaying cognitive impairments using prevention and intervention strategies.
